# Inhibition of cysteine protease disturbs the topological relationship between bone resorption and formation in vitro

**DOI:** 10.1007/s00774-023-01489-w

**Published:** 2024-02-20

**Authors:** Sayaka Ono, Naoki Tsuji, Tomoaki Sakamoto, Shuya Oguchi, Takashi Nakamura, Kazuto Hoshi, Atsuhiko Hikita

**Affiliations:** 1 Department of Sensory and Motor System Medicine, Graduate School of Medicine, The University of Tokyo, Tokyo, 113-8655 Japan; 2 Department of Tissue Engineering, The University of Tokyo Hospital, Tokyo, 113-8655 Japan; 3 Department of Oral-Maxillofacial Surgery, and Orthodontics, The University of Tokyo Hospital, Tokyo, 113-8655 Japan; 4 Department of Biochemistry, Tokyo Dental College, Tokyo, 101-0061 Japan

**Keywords:** Bone remodeling, Coupling, Two-photon microscopy, Cathepsin K, Osteoclast, Osteoblast

## Abstract

**Introduction:**

Osteoporosis is a global health issue. Bisphosphonates that are commonly used to treat osteoporosis suppress both bone resorption and subsequent bone formation. Inhibition of cathepsin K, a cysteine proteinase secreted by osteoclasts, was reported to suppress bone resorption while preserving or increasing bone formation. Analyses of the different effects of antiresorptive reagents such as bisphosphonates and cysteine proteinase inhibitors will contribute to the understanding of the mechanisms underlying bone remodeling.

**Materials and Methods:**

Our team has developed an in vitro system in which bone remodeling can be temporally observed at the cellular level by 2-photon microscopy. We used this system in the present study to examine the effects of the cysteine proteinase inhibitor E-64 and those of zoledronic acid on bone remodeling.

**Results:**

In the control group, the amount of the reduction and the increase in the matrix were correlated in each region of interest, indicating the topological and quantitative coordination of bone resorption and formation. Parameters for osteoblasts, osteoclasts, and matrix resorption/formation were also correlated. E-64 disrupted the correlation between resorption and formation by potentially inhibiting the emergence of spherical osteoblasts, which are speculated to be reversal cells in the resorption sites.

**Conclusion:**

These new findings help clarify coupling mechanisms and will contribute to the development of new drugs for osteoporosis.

**Supplementary Information:**

The online version contains supplementary material available at 10.1007/s00774-023-01489-w.

## Introduction

Osteoporosis, a major public health problem for over 200 million people around the world [1], is a skeletal condition characterized by low bone density and microstructural degradation of bone tissue, which together increase the risk of fracture [2]. Drugs such as bisphosphonates (BPs), denosumab, vitamin preparations, calcium preparations, parathyroid hormone (PTH) agents, and anti-sclerostin antibodies are used to treat osteoporosis [3]. Although BPs are the most widely used agents in the treatment of osteoporosis [4], the suppression of bone resorption by a BP also leads to a subsequent decrease in bone formation. Decreased bone turnover in bone metabolism leads to a decline in bone quality and increases the risk of atypical fractures [5]. Bisphosphonates were also suggested to be involved in the development of medication-related osteonecrosis of the jaw [6].

The limited duration of a regimen for bone-forming agents such as PTH and anti-sclerostin antibodies poses several issues, including the decrease in bone density after the treatment period and the constraints imposed by the treatment schedule. There is a strong demand for the development of new medications for osteoporosis that can overcome these limitations.

Osteoclasts resorb bone by producing cathepsin K, a powerful cysteine proteinase that disassembles collagen [7]. When cathepsin K is inhibited by E-64, a cysteine proteinase inhibitor, bone resorption is suppressed [8, 9]. In cathepsin K knockout mice, bone resorption is inhibited and bone formation is enhanced due to an increase in sphingosine 1-phosphate (S1P) produced by osteoclasts [10].

Based on these findings, the cathepsin K inhibitor odanacatib (ODN) was developed for the treatment for osteoporosis. ODN reduced bone turnover in the lumbar vertebrae of surgically menopausal cynomolgus monkeys, suppressing trabecular bone resorption and increasing bone mass [11]. The addition of ODN to human osteoclasts significantly reduced the release of CTX-I [12]. Clinical trials of ODN reported increases in bone density, reductions in fracture risk, and the promotion of bone formation [13]. Although the development of ODN was discontinued due to an increased risk of stroke observed in a phase 3 trial [14], the findings obtained are valuable for future studies. ODN and BPs are both bone resorption inhibitors, but they have different impacts on subsequent bone formation. Detailed analyses of the effects of these drugs on bone remodeling could enhance our understanding of bone metabolism and thus contribute to the development of novel treatments for osteoporosis.

However, it is challenging to analyze bone remodeling at the cellular level in vivo. Bone remodeling is a process that spans weeks to months, and its analysis requires observations of bone resorption followed by bone formation at the same site. Histological analyses and bone morphometry provide information from a single time point, and it is impossible to analyze the spatiotemporal relationship between bone resorption and formation, as well as cell interactions. Long-term observation is challenging with in vivo imaging, due to the invasiveness of the observation process. Moreover, the limited vertical resolution in the imaging of the bone marrow makes it difficult to accurately evaluate the bone matrix.

To solve these problems, our team developed an in vitro system that reconstructs the bone cell network involving osteoclasts, osteoblasts, and osteocytes within the mineralized nodule, enabling the visualization of bone modeling and remodeling phenomena by 2-photon microscopy [15]. We used this system in the present study to investigate the effects of E-64 and zoledronic acid (ZOL), a bisphosphonate, on bone remodeling over a long term.

## Materials and methods

### Mice

All animal experiments were approved by the Animal Experiment Committee of the Graduate School of Medicine of the University of Tokyo (#P15-019 and #P19-114). C57BL/6-Tg(CAG-EGFP). C14-Y01-FM131Osb mice in which all cells express enhanced green fluorescent protein (EGFP) under the control of the CAG promoter (EGFP mice) were purchased from the Japan RIKEN BioResource Center (Ibaraki, Japan). B6.Cg-Gt(ROSA)26Sortm14(CAG-tdTomato) Hze/J mice (Ai14) [ROSA26-tdTomato mice purchased from The Jackson Laboratory (Bar Harbor, ME, USA)] were crossed with Ctsk-Cre mice expressing cre recombinase under the control of the cathepsin K promoter [16] (provided by Dr. Shigeaki Kato, Fukushima Medical University, Fukushima, Japan).

### Preparation of primary osteoblasts

Primary osteoblasts were isolated from EGFP mice as described [15, 17, 18] with a few modifications. Briefly, the calvaria were harvested from newborn mice aged 0–5 days, and the soft tissues attached to their surface were removed with cell scrapers. Parietal bones were collected and incubated in 3.5 mL of Minimum Essential Medium-α (MEM-α; Thermo Fisher Scientific) supplemented with 100 µg/mL of collagenase P (Roche Diagnostics), and 88 µL of 0.05% trypsin/EDTA (Thermo Fisher Scientific) per calvaria at 37 °C for 20 min with a shaking water bath. The parietal bones were then chopped into small pieces and incubated in 800 µL MEM-α supplemented with 200 µg/mL collagenase P and 20 µL of 0.05% trypsin/EDTA per calvaria for 15 min at 37 °C.

After the incubation, the bone pieces were washed twice with MEM-α, and cultured with a new culture medium (MEM-α supplemented with 15% FBS, 100 U/mL penicillin, and 100 µg/mL streptomycin) for 5–6 days, and cells that grew out of the bone pieces were collected.

### Preparation of bone marrow macrophages

Bone marrow cells were collected from the tibia, femur, and humerus of Ctsk-Cre × ROSA26-tdTomato mice as described [15, 18, 19]. The bone marrow cavity was flushed with MEM-α, and the solution was centrifuged at 430*g* for 5 min. Cells were treated with Tris-NH_4_Cl for 2 min to lyse red blood cells and then centrifuged at 430*g* for 3 min after the addition of 1 mL of FBS [20]. After an overnight culture with MEM-α supplemented with 10% FBS, 100 U/mL penicillin, 100 µg/mL streptomycin, and 10 ng/mL macrophage colony-stimulating factor (M-CSF) (R&D Systems), floating cells were collected.

### Cell culture for the in vitro reconstruction system

Osteoblasts from EGFP mice were cultured in 60-mm dishes at 4 × 10^5^ to 5.5 × 10^5^ cells/dish with the culture medium until they reached confluence at 3–5 days. To induce osteoblast differentiation, the cells were cultured in the osteoblast differentiation medium [MEM-α supplemented with 10% FBS, 100 U/mL penicillin, 100 µg/mL streptomycin, 100 µg/mL L(+)-ascorbic acid (Fujifilm Wako), and 5 mM β-glycerophosphate disodium salt hydrate (Sigma-Aldrich)] with 0.1 µM cFMS Receptor Inhibitor II (Santa Cruz Biotechnology). Calcified nodules were formed after 4–6 weeks of differentiation culture, and 2 × 10^6^ bone marrow macrophages from Ctsk-Cre × ROSA26-tdTomato mice were added. They were co-cultured with the co-culture medium (MEM-α supplemented with 10% FBS, 100 U/mL penicillin, 100 µg/mL streptomycin, 10^−6^ M prostaglandin E2 [Sigma-Aldrich], and 10^−8^ M 1α, 25-dihydroxy vitamin D3 [Sigma-Aldrich] with or without 10 µM E-64 or 1 µM ZOL) for 2 weeks, and with osteoblast differentiation medium for 3 weeks. E-64 or ZOL were continuously administered to the medium from weeks 0 to 5.

To count osteoclast nuclei in co-culture conditions, we stained cells with 5 µg/mL of Cellstain-Hoechst 33342 solution after 2 weeks of co-culture. Stained nuclei were counted manually.

### Image acquisition for in vitro reconstruction system

Images were acquired as described [15, 17, 18], with slight modifications. Cells and matrices in the in vitro reconstruction system were observed by 2-photon microscopy every week (7 days ± 1 day) with a multiphoton confocal microscopy system (A1R + MP, Nikon, Tokyo) with a titanium-sapphire laser (wavelengths: 680–1050 nm, repetition rate: 80 MHz, pulse width: 70 fs; Mai Tai eHP, Spectra-Physics), a water-immersion objective lens (numerical aperture: 1.1; CFI75 Apo 25 × WMP, Nikon) and the following emission filters: 492-nm short-pass for second harmonic generation (SHG), 525/50-nm band-pass for EGFP, and 575/25-nm band-pass and 629/56-nm band-pass for tdTomato.

The SHG from collagen fibers, the emissions from EGFP expressed in osteoblasts and osteocytes, and the tdTomato emission from osteoclasts were all observed using excitation light at a wavelength of 930–950 nm. Three-dimensional images were taken at *Z*-steps of 1 µm. Some zoom-up images were captured at *Z*-steps of 0.5 µm. The observation began at co-culture week 0 and ended at co-culture week 5. The same position was observed weekly. Images were taken from six regions in each dish. After each observation, fresh medium was added after the cells had been rinsed twice with PBS to prevent contamination. Three series of experiments were conducted.

### Data analysis

The software program NIS Elements ver. 4.30.00 (Nikon) used a median filter and local contrast image processing to preprocess all of the images, but the images of sites with extensively saturated tdTomato signals were omitted from the quantitative analysis. Accordingly, 3–6 regions were analyzed in each dish. The acquired data’s XYZ misalignment was adjusted manually. The preprocessed images were examined with Imaris ver. 8.3.1 software (Oxford Instruments, Abingdon, UK). Sixteen ROIs were created out of a single field of view. To calculate the average volume of osteoclasts (Fig. 4c), the volume of osteoclasts with a volume over 2000 µm^3^ at R1 and R2 was calculated to omit mononuclear cells with typical diameter of 15–22 μm [21] and cell debris. An Imaris Reader was used for projecting all of the data into MATLAB R2023a (MathWorks) for the quantitative data.

### Statistical analyses

All data are expressed as the mean ± SE (standard error). MATLAB R2023a (MathWorks) was used to plot the data and perform the statistical analyses. The Lilliefors test and the Kolmogorov–Smirnov test were used to determine whether the data were normally distributed. Spearman’s rank correlation coefficients were calculated to analyze the correlations between values. The rho values were categorized as follows: 0 ≤|*R*|< 0.2 = negligible correlation, 0.2 ≤|*R*|< 0.4 = weak correlation, 0.4 ≤|R|< 0.6 = moderate correlation, and 0.6 ≤|*R*|< 0.8 = strong correlation. Probability (*p*) values < 0.05 were considered significant.

## Results

### In vitro reconstitution system of bone remodeling

The in vitro reconstitution system was prepared by co-culturing bone marrow macrophages from Ctsk-Cre × ROSA26-tdTomato mice with bone nodules formed by osteoblasts from EGFP mice. The isolated osteoblasts from EGFP mice were confirmed to be alkaline phosphatase (ALP)-positive (Suppl. Fig. S1a), and the isolated bone marrow cells from Ctsk-Cre × ROSA26-tdTomato mice were confirmed to differentiate into tartrate-resistant acid phosphatase (TRAP)-positive osteoclasts (Suppl. Fig. S1b).

We first optimized the concentrations for E-64 and ZOL. At a 10 µM or more concentration of E-64, resorption pits decreased as described [9] (Suppl. Fig. S2a, b). The cytotoxicity of E-64 was confirmed through the loss of cellular autofluorescence tdTomato signal due to cell death, and found no significant difference (Suppl. Fig. S2c). From these results, we determined to use 10 µM E-64, as previously described [9]. At ZOL concentrations above 1 µM, almost no osteoclast-like TRAP-positive cells were observed (Suppl. Fig. S2d, e), as reported [22].

To compare the effects of E-64 and ZOL on osteoclast differentiation, we treated osteoclasts with 10 µM E-64 or 1 µM ZOL (Suppl. Fig. S3a). There was no significant difference in the number of TRAP-positive cells with actin rings between the control and E-64 groups, whereas the ZOL-treated osteoclasts showed a significant decrease compared to cells of other groups (Suppl. Fig. S3b). The number of nuclei per osteoclast decreased significantly in both the E-64 and ZOL groups (Suppl. Fig. S3c, d).

To evaluate the effects of E-64 and ZOL on the nodule formation, 10 µM E-64 or 1 µM ZOL was added to the osteoblast differentiation culture, and alizarin red staining was performed after 28 days (Suppl. Fig. S3e). As a result, the ZOL group showed a significant decrease (Suppl. Fig. S3f).

**Fig. 1:**
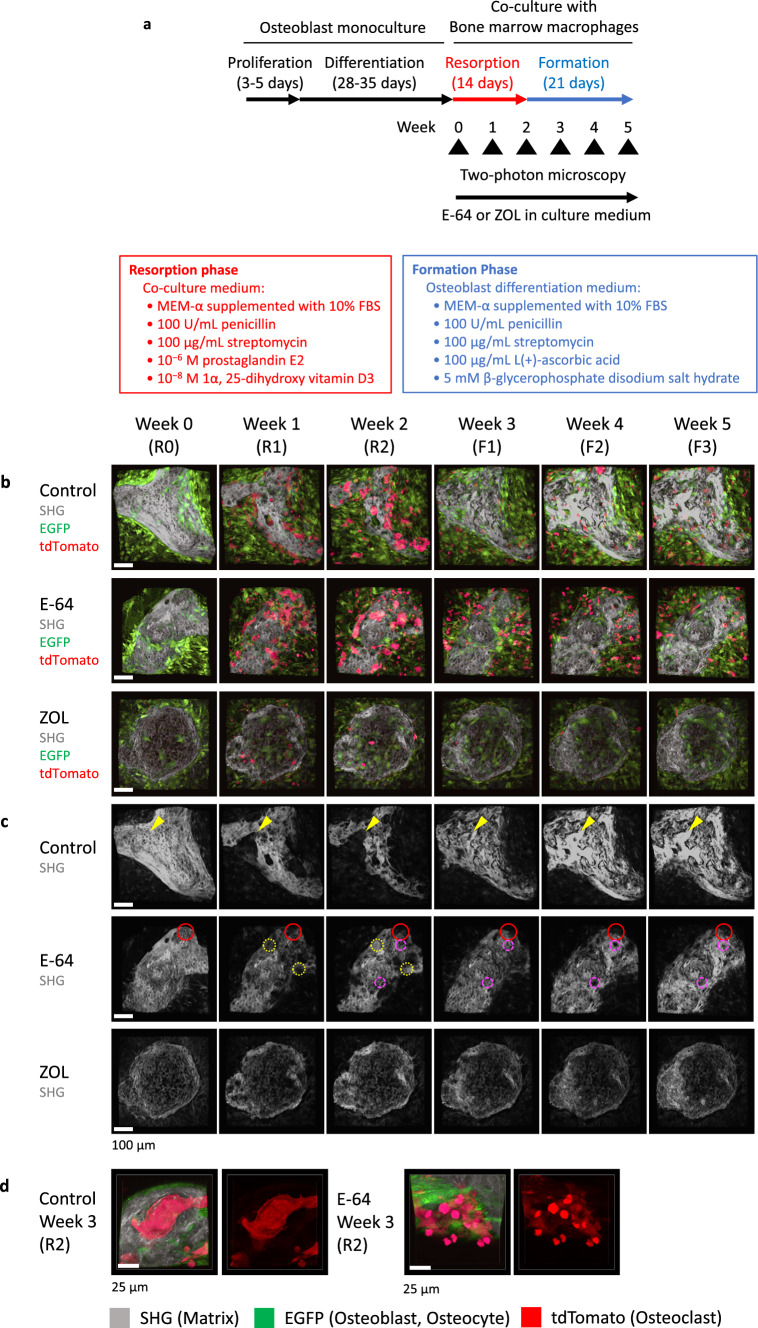

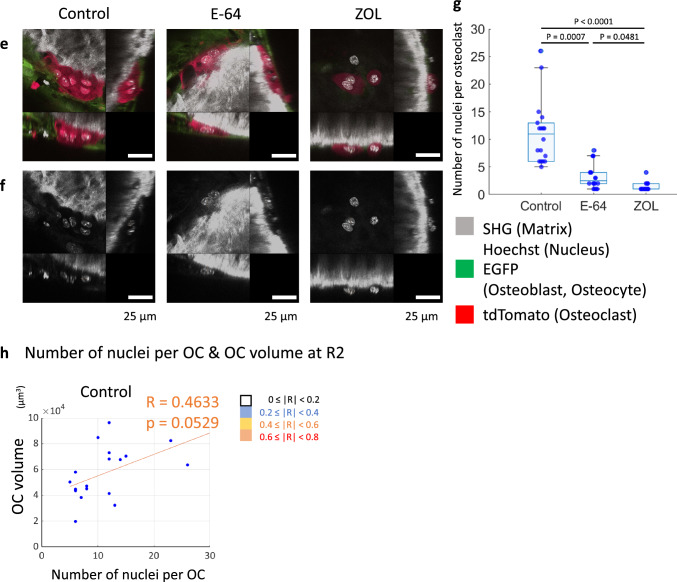
The in vitro reconstitution system of bone remodeling. **a** Schema of the experimental design. Weeks 0, 1, 2, 3, 4, and 5 are referred to as R0, R1, R2, F1, F2, and F3 hereafter. **b**,** c** Volumetric views of 3D data acquired by 2-photon microscopy. Maximal impression images shown by IMARIS are presented. **b** Volumetric views of all channels. Gray: second harmonic generation (SHG; collagen). Green: EGFP (osteoblasts). Red: tdTomato (osteoclasts). **c** Volumetric views of the SHG channel. To make the images more visible, the brightness of each image was modified using look-up tables (LUTs) and the same values. Scale bar: 100 µm. Yellow arrowheads in the control group: representative resorption pits that were filled with the new matrix created. Dotted yellow circles in E-64: sites at which resorption occurred in the 1st week of the resorption phase and formation occurred in the 2nd week. Dashed magenta circles in E-64: sites at which formation occurred in the 1st week of the formation period, resorption occurred in the 2nd week, and formation occurred again in the 3rd week. **d** Zoom-up images of osteoclasts shown by NIS-elements AR software (Nikon). **e**, **f** Orthogonal views of osteoclasts at R2. The nuclei were stained with Hoechst 33342. The images are via NIS-elements AR. Scale bar: 25 µm. **e** Volumetric views of all channels. Gray: SHG. Green: EGFP (osteoblasts). Red: tdTomato (osteoclasts). **f** Volumetric views of the SHG channel. **g** Quantification of the number of nuclei per osteoclast in panels **e** and **f**. Data were analyzed by the Kruskal–Wallis test followed by the Steel–Dwass test. Six fields of each dish were observed, and the experiment was carried out three times (*n* = 18). Data are mean ± SE. **h** A scatterplot between the number of nuclei per osteoclast and the osteoclast volume (R2). Spearman’s rank correlation coefficients (*R*) and *p* values. *n* = 18

To evaluate the effects of E-64 and ZOL on bone remodeling, we added them to the in vitro reconstitution system. The observations were started at the end of the differentiation culture of osteoblasts (hereinafter referred to as R0). After the first observation, bone marrow macrophages from Ctsk-Cre × ROSA26-tdTomato mice were co-cultured with differentiated osteoblasts (Fig. 1a). The bone marrow macrophages turn red when cathepsin K was expressed during osteoclastogenesis. SHG, which can detect collagen without fluorescence labeling, was used to detect bone nodules formed by osteoblasts.

Resorption pits were formed in the nodules of the control group during the resorption phase (week 0 [R0] to week 2 [R2]) and were later filled with freshly generated matrix in the following formation phase (week 3 [F1] to week 5 [F3], (Fig. 1b, c, yellow arrowheads). The resorption pits in the E-64 group were not as prominent as those of the control group (Fig. 1c). In addition, the synchronicity of the resorption-formation cycles was disturbed by E-64. For example, in some areas, matrix was resorbed at R1 and formed at R2 (Fig. 1c, yellow circles). In other regions (Fig. 1c, magenta circles), the formation occurred at F1, followed by resorption and formation at F2 and F3, respectively. Some resorption pits were not filled (Fig. 1c, red circles). In the ZOL group, changes in the matrix were not as obvious as in the other groups (Fig. 1b, c).

In the control group, multinucleated large osteoclasts appeared at resorption pits during the resorption period, and the matrix was resorbed in a large area (Fig. 1d–f). In the E-64 group, some osteoclasts were detached from the matrix as mentioned in an earlier study [23] (Fig. 1d). Osteoclasts with few nuclei formed small resorption pits (Fig. 1e, f). The number of nuclei was significantly decreased by adding E-64 or ZOL to the co-culture of osteoclasts and osteoblasts, as was the case in the monoculture of osteoclasts (Fig. 1e–g, Suppl. Fig. S3c, d). The number of nuclei per osteoclast and the osteoclast volume (R2) showed a tendency of moderate correlation in the control group (Fig. 1h).

We also confirmed the deposition of ZOL on calcified nodules and its uptake by osteoclasts using fluorescence-labeled ZOL in the in vitro reconstitution system (Suppl. Fig. S3g–n).

### The changes in the overall matrix, osteoclasts, and osteoblasts volume

**Fig. 2:**
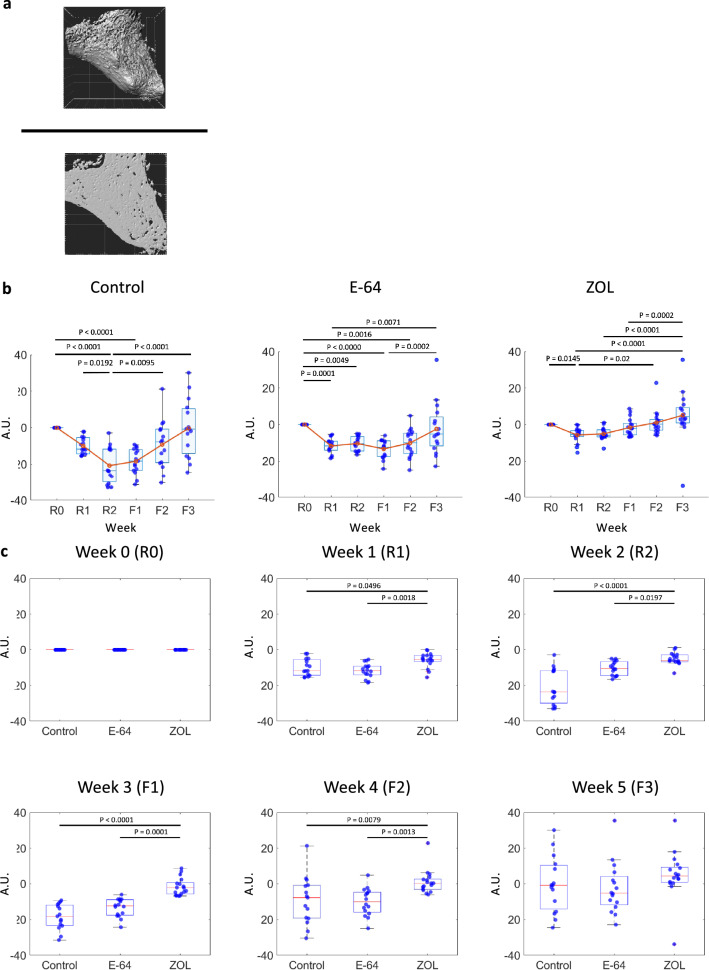

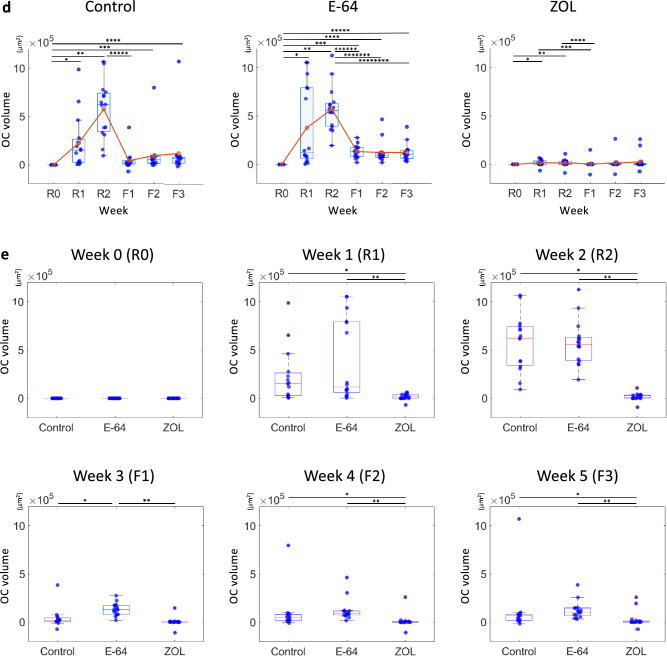

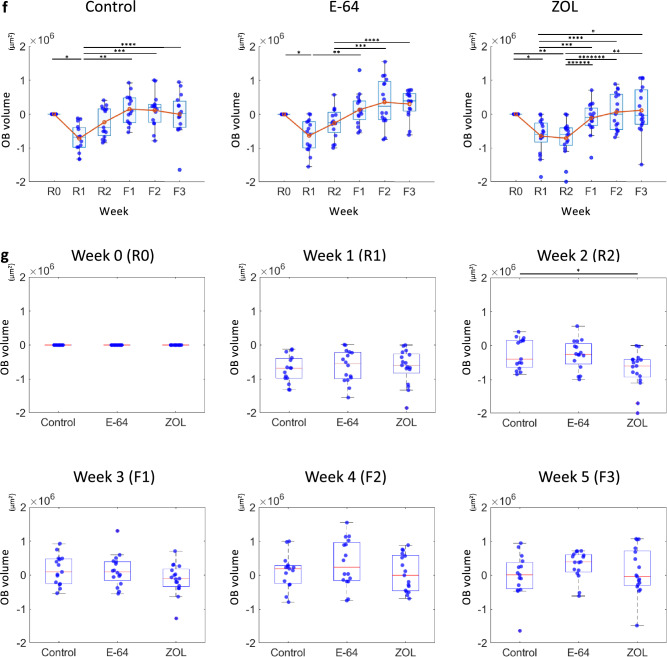
Analyses of SHG, tdTomato, and EGFP volumes. **a** Surface rendering of the SHG positive area (upper panel). The value of the area of maximum SHG projection at week 0 (lower panel) was used to adjust the SHG volumes. Temporal changes in the SHG (**b**), tdTomato (**d**), and EGFP (**f**) volume for each group. Data are mean ± SE. The Friedman test followed by Bonferroni adjustment was used to determine the significance of differences between the values at each time point. **c**, **e**,** g** Comparisons of the three groups’ values at each time point. Data are mean ± SE and were analyzed by the Kruskal–Wallis test followed by the Steel–Dwass test. Control: *n* = 15, E-64: *n* = 16, ZOL: *n* = 17. **c** SHG, **e** tdTomato, **g** EGFP

Surface rendering was performed for SHG-, EGFP-, and tdTomato-positive regions for the measurement of the temporal changes in matrix, osteoblasts, and osteoclasts, respectively. The volume and surface area were then calculated. We used the projected area of the SHG at R0 to modify the volume of the SHG, and differences between the values at each time point and those at R0 were calculated (Fig. 2a). First, the temporal changes in the SHG volume were evaluated in each group. The SHG volumes at R2 in the control group were significantly lower than those at R0, R1, F2, and F3, indicating the significant reduction and formation of matrix during the resorption and formation phases, respectively. The SHG volumes at R1, R2, F1, and F2 of the E-64 group were significantly lower than those at R0, in a pattern different from that of the control group. In the ZOL group, the SHG volumes at R1 were significantly lower than at R0 (Fig. 2b).

The differences in the SHG volumes among the three groups at the same time points were also analyzed. The SHG volume in the ZOL group was significantly larger than those of other groups at R1, R2, F1, and F2. There was no significant difference between each group at F3 (Fig. 2c).

We also analyzed the changes in the overall osteoclasts (Fig. 2d, e) and osteoblasts volume (Fig. 2f, g). The osteoclast volume in the E-64 group was significantly larger than those of other groups at F1, and those in the ZOL group were lower than those in the other groups at R1, R2, F2, and F3 (Fig. 2e). The EGFP volume in the ZOL group was significantly lower than those of the control groups at R2 (Fig. 2g).

### The regional changes in the matrix volume

**Fig. 3:**
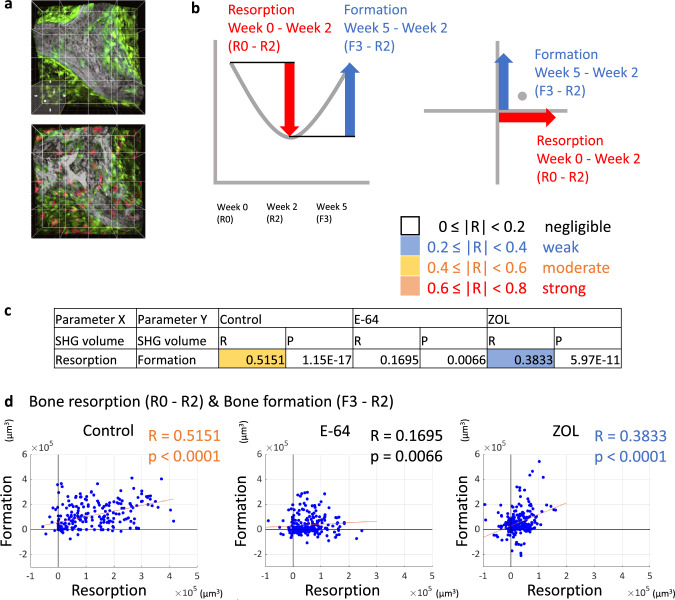
Analysis of regional changes in the SHG volume. **a** Sixteen regions of interest (ROIs) in one field of view. **b** Explanatory diagrams for the values of bone resorption and bone formation, and the scatterplots. Changes in the values during the resorption phase (week 0 [R0] to week 2 [R2]) and the formation phase (week 2 [R2] to week 5 [F3]) are plotted. **c**, **d** Spearman’s rank correlation coefficients (R) and *p* values (**c**) and scatterplots (**d**) for bone resorption and bone formation. Control: *n* = 240, E-64: *n* = 256, ZOL: *n* = 272

One field of view was divided into 16 ROIs for the analysis of regional changes in the parameters for matrices, osteoclasts, and osteoblasts, and correlations among them [5] (Fig. 3a). To investigate the quantitative and topological correlations between bone resorption and formation, we analyzed the amount of change in the SHG volume during the resorption phase and formation phase in each ROI (Fig. 3b). Bone resorption and bone formation moderately correlated in the control group, indicating that the resorption and formation of the matrix were balanced in each region. In the ZOL group, the corresponding correlations between values were weak. The correlations were negligible in the E-64 group, suggesting that the topological relationship between resorption and formation was disturbed by the inhibition of cysteine proteinases (Fig. 3c, d).

### The correlations between matrix changes and osteoclast volumes

To investigate the effect of osteoclasts on the matrix, we calculated the osteoclast volumes in the ROIs and their correlations with bone resorption/formation. The osteoclast volume peaked at R2 in the control and E-64 groups, and negligible change was observed in the ZOL group, indicating the suppression of osteoclastogenesis (Fig. S4a).

**Fig. 4:**
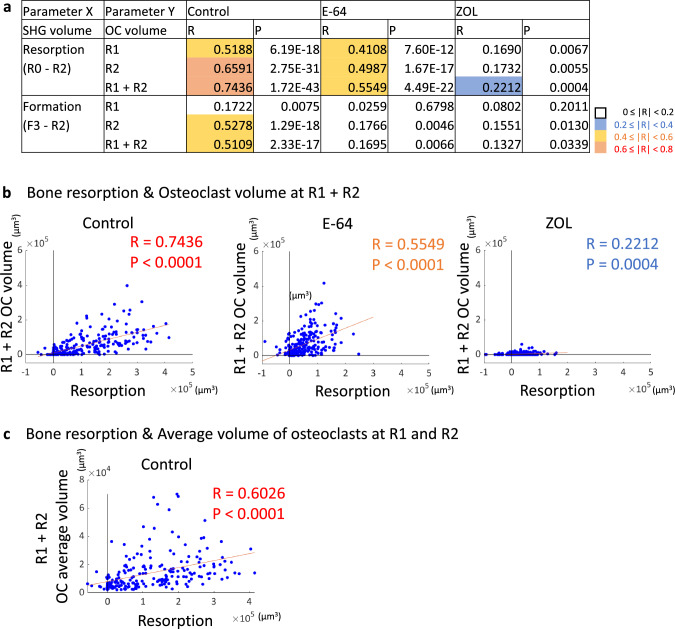
Analysis of the correlations between the matrix changes and the osteoclast volume. **a** Correlations between the changes in the matrix volume and the changes in the osteoclast volume. Spearman’s rank correlation coefficients (R) and *p* values. **b** Scatterplots between bone resorption and the cumulative osteoclast volume (R1 + R2). Control: *n* = 240, E-64: *n* = 256, ZOL: *n* = 272. **c** A scatterplot between bone resorption and the total volume of osteoclasts with a volume over 2000 µm^2^ at R1 and R2, divided by the number of ROI. Spearman’s rank correlation coefficients (*R*) and *p* values. Control: *n* = 240

The analysis of the correlations revealed that the osteoclast volume at R2 and the cumulative osteoclast volume (R1 + R2) were strongly correlated with bone resorption in the control group. The correlations were reduced to moderate ones in E-64 group, and almost disappeared in ZOL group (Fig. 4a, b). In the control group, there was a strong correlation between bone resorption and average volume of osteoclasts at R1 and R2 (Fig. 4c).

### The correlations between matrix changes and osteoblast volumes

The osteoblast volumes in each ROI were calculated and their correlations with bone resorption/formation were examined. The osteoblast volume was lowest at R1 in the control and E-64 groups, whereas in the ZOL group, it was lowest at R2 (Suppl. Fig. S5a).

**Fig. 5:**
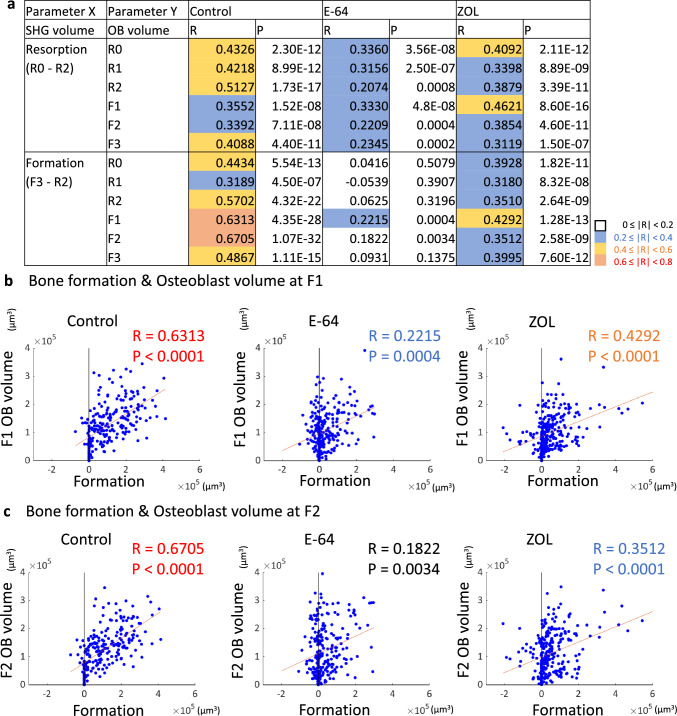
Analysis of the correlations between matrix changes and the EGFP volume. **a** Correlations between bone resorption/formation and the osteoblast volume. Spearman’s rank correlation coefficients (*R*) and *p* values are presented. **b**, **c** Scatterplots between bone formation and the osteoblast volume at F1 (**b**), and F2 (**c**). Control: *n* = 240, E-64: *n* = 256, ZOL: *n* = 272

When the correlations between bone formation and osteoblast volume were analyzed, the osteoblast volumes at F1 and F2 showed a strong correlation with bone formation in the control group. On the other hand, only a moderate correlation was observed between bone formation and OB volume at F1 in the ZOL group. Furthermore, in the E-64 group, only the osteoblast volume at F1 was weakly correlated with bone formation (Fig. 5a–c).

### The correlations between matrix changes and osteoblast sphericity

**Fig. 6:**
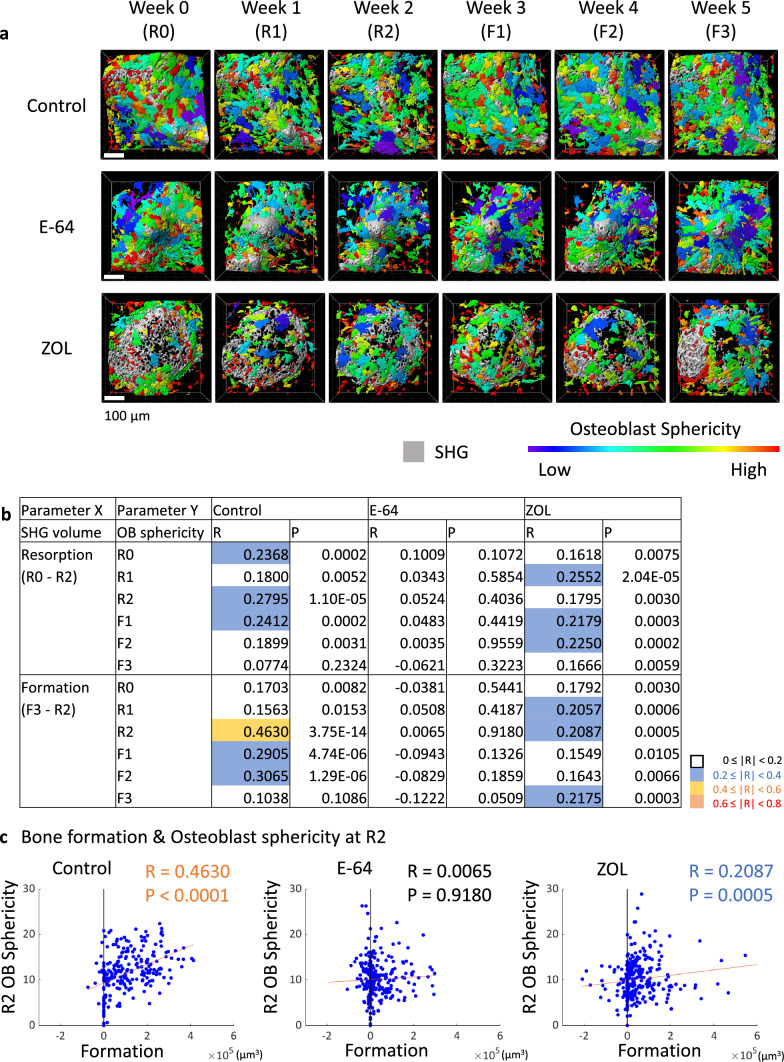
Analysis of the correlations between bone resorption/formation and osteoblast sphericity. **a** Surface rendering of SHG and EGFP shown by the Imaris software. The EGFP-positive area is shown with a heatmap indicating the extent of sphericity. Scale bar: 100 µm. **b** Correlations between bone formation/resorption and osteoblast sphericity. Spearman’s rank correlation coefficients (R) and *p* values are presented. **c** Scatterplots between bone formation and the osteoblast sphericity at R2. Control: *n* = 240, E-64: *n* = 256, ZOL: *n* = 272

Cuboidal osteoblasts with high sphericity are suggested to be active osteogenic cells [15, 17]. We evaluated the correlations between bone resorption/formation and the sphericity of osteoblasts. From the surface rendering of the SHG and EGFP with heatmaps (Fig. 6a) and the changes in the osteoblast sphericity of each ROI over time (Suppl. Fig. S6a), we did not observe distinct patterns. The analysis revealed that the sphericity of osteoblasts at R2 and bone formation were moderately correlated, suggesting the osteoblasts with relatively high sphericity that existed at the end of the resorption phase affected the subsequent bone formation (Fig. 6b, c).

### The correlations between the osteoblast volume and osteoclast volume

**Fig. 7:**
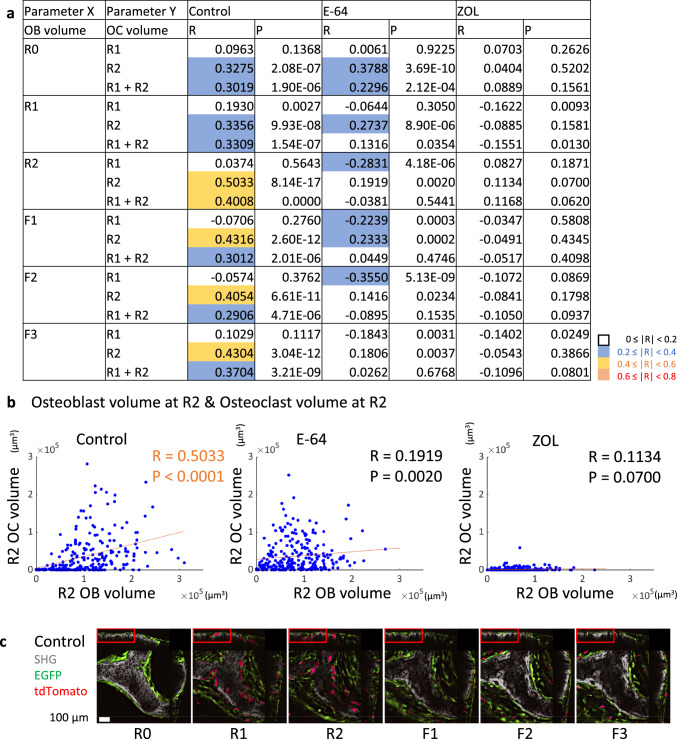

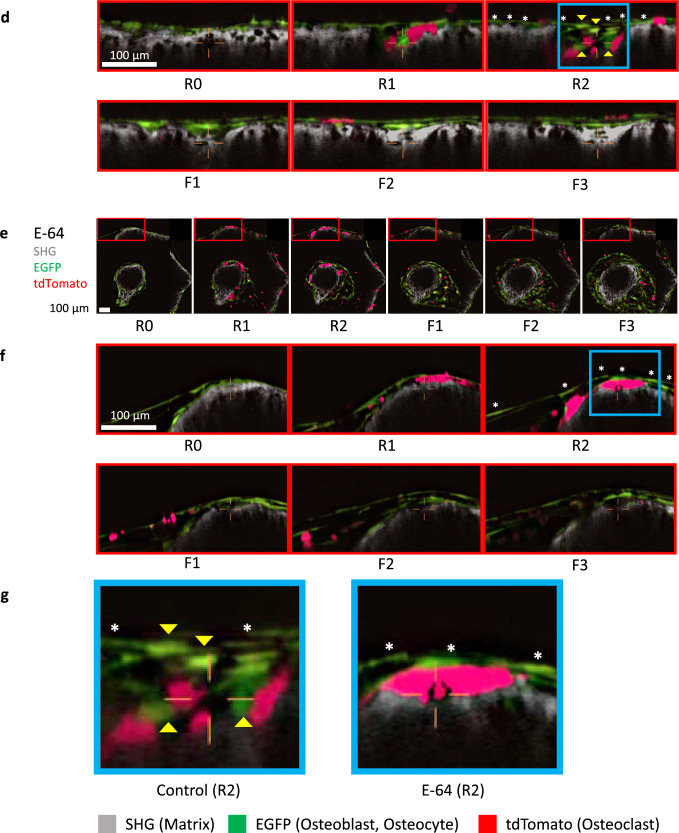
Analysis of the correlations between the osteoblast volume and osteoclast volume. **a** Correlations between the osteoblast volume and osteoclast volume. Spearman’s rank correlation coefficients (*R*) and *p* values are presented. **b** Scatterplots between the osteoblast volume at R2. Control: *n* = 240, E-64: *n* = 256, ZOL: *n* = 272. **c–g** Orthogonal views of resorption pits in the control and E-64 groups. **c** Orthogonal views of the control (see Fig. 1b). The images were revealed by NIS-elements AR software. **d** Cropped pictures of panel **c** are indicated by red boxes. Yellow arrowheads: spherical osteoblasts. *Flat osteoblasts. **e** Orthogonal views of E-64 (see Fig. 1b). The images are by NIS-elements AR. **f** Cropped pictures of panel **e** are indicated by red boxes. *Flat osteoblasts. To make images more visible, the brightness of each image was modified using LUTs and the same values. Scale bar: 100 µm. **g** Cropped pictures of panels **d** and** f**, respectively, as indicated by blue boxes. Yellow arrowheads: spherical osteoblasts. *Flat osteoblasts

Finally, we analyzed the correlation between osteoblasts and osteoclasts (Fig. 7a, b). In the control group, the osteoclast volume at R2 was moderately correlated with the osteoblast volumes at R2, F1, F2 and F3, and the cumulative osteoclast volume (R1 + R2) also showed a moderate correlation with the osteoblast volume at R2, suggesting the existence of interactions between these two lineages of cells. However, only weak or negligible correlations were observed in the E-64 and ZOL group (Fig. 7a, b).

### Temporal changes in resorption pits

Representative findings that confirm the analysis results with images are provided as Fig. 7c–g. In the control group, osteoclasts expressing tdTomato appeared during the resorption phase and around resorbed matrix. Osteoblasts with high sphericity were observed below the canopy-like structure composed of flat osteoblasts and around the osteoclasts in the resorption pit. In the subsequent formation phase, the resorption pits were then filled specifically. In the adjacent bone surface covered with flat osteoblasts, no resorption or formation was observed (Fig. 7d, g). In the E-64 group, red osteoclasts appeared during the resorption phase, and a shallow resorption pit was formed. The resorption site was covered with flattened osteoblasts. Migrations of spherical osteoblasts were not observed. In addition, the resorption site was not filled by the newly synthesized matrix (Fig. 7f, g).

## Discussion

We compared the effects of different bone resorption inhibitors on bone remodeling using an experimental system that replicates the network of osteoblasts, osteoclasts, and osteocytes with the extracellular bone matrix in vitro. In the control group, the resorption and refilling of matrix were clearly indicated by the disappearance and reappearance of the SHG signal at the same regions (Figs. 1b, c, 7d). The quantification of SHG volume also demonstrated the balanced decrease and increase in the matrix (Fig. 2b). The increase in the tdTomato-positive region during the formation phase represents the maturation of osteoclasts (Figs. 1b, 2d, e), accompanied with increase in lysosomes [24] and mitochondria [25] to degrade intracellular and extracellular material [24] and aid in the cell’s recovery [25], respectively. On the other hand, osteoclasts decreased in the formation phase (Figs. 1b, 2d, S4a), probably due to osteoclast apoptosis. However, we have not examined the mode of cell death. In this in vitro system with limited nutrition, it is possible that not only apoptosis but also necrosis due to energy depletion may be involved.

In contrast, in the ZOL group, temporal changes in matrix were not as obvious as those in other groups (Fig. 1b, c). The quantification also revealed that bone resorption and the subsequent formation were suppressed (Fig. 2b, c). Osteoclasts were rarely seen (Figs. 1b, 2d, e), which agreed with previous reports showing that bisphosphonates cause the apoptosis of osteoclasts [2, 26] and inhibit the differentiation and activity of osteoclasts [27]. It has been proposed that BPs reduce the bone remodeling rate indirectly by suppressing bone resorption, while a study of 2021 demonstrated that BPs directly affect the osteoblasts [28]. Although our present data are not sufficient to determine whether BPs affected the osteoblasts directly or indirectly, the suppression of matrix resorption and subsequent formation by a BP was replicated in our system.

In the E-64 group, the resorption pits seemed smaller and shallower than those in the control group, and refilling occurred in an unsynchronized manner (Figs. 1b, c, 8f, g). Although there was no significant difference compared to the control group, the amount of SHG reduction was smaller (Fig. 2b, c). E-64 decreases the resorption amount and resorption area by osteoclasts in vitro [29]. The treatment of osteoclasts with cysteine protease inhibitors resulted in the formation of abnormal pits filled with demineralized but un-degraded matrix on the surface of ivory slices [30]. Cysteine proteinase inhibitors were also reported to suppress osteoclastogenesis [21]. Our present findings also demonstrated the suppression of multinucleation of osteoclasts by E-64 treatment (Fig. 1e, g, Suppl. Fig S3c, d). Interestingly, small osteoclasts tended to persist in the formation phase in the E-64 group (Figs. 1b, 2d, e). Because larger osteoclasts are more prone to undergo apoptosis than smaller ones [31], suppression of multinucleation may have suppressed the apoptosis of osteoclasts in E-64 group. Another possible explanation is that suppression of bone resorption that consumes large amounts of adenosine triphosphate produced by glycolysis and oxidative phosphorylation [32] may have suppressed apoptosis. Regarding bone formation, cysteine protease inhibitors have been shown to suppress osteoblast proliferation, differentiation, and functions [29, 33]. This study also revealed the suppression of the mineralized nodule formation, though not significantly (Suppl. Fig. S3e, f). The E-64 treatment resulted in a matrix volume that was comparable to that of the control group at F3, by reductions of both resorption and formation (Fig. 2b, c).

When we analyzed each parameter in the 16 ROIs per field of view, we observed that resorption and formation were moderately correlated in the control group as previously [18], indicating the topological and quantitative coordination between resorption and formation (Fig. 3c, d). The osteoclast volume and osteoblast volume were strongly correlated with both resorption and formation, while the osteoclast volume and osteoblast volume were moderately correlated (Figs. 4a, 5a, 7a). In addition, there were correlations between the number of nuclei and volume of osteoclasts, and the average volume of osteoclast and bone resorption capacity in the control group (Fig. 4c, 1h), which agreed with a previous study indicating the correlation of number of nuclei and resorptive activity of osteoclasts [34].

On the other hand, in the ZOL group, resorption and formation correlated only weakly, and the correlation among osteoclasts, osteoblasts, and matrix was also reduced. Moreover, only negligible correlation was detected between resorption and formation in the E-64 group, suggesting the loss of the topological and quantitative coordination. This result may not be attributable only to the suppression of matrix formation by osteoblasts, because the effect on the nodule formation was greater in the ZOL group than in the E-64 group (Suppl. Fig. S3e, f). In the E-64 group, the correlation between the osteoclast volume and resorption was preserved to some extent, whereas the osteoclast volume and formation seldom correlated (Fig. 4a).

The correlations between matrix resorption/formation and the osteoblast volume at some time points were moderate in the ZOL group, while only weak correlations were observed in the E-64 group (Fig. 5a). The correlations between the osteoclast volume and osteoblast volume were negligible in the ZOL group, although there were some weak correlations in E-64 group (Fig. 7a). These data suggested that the link between osteoblasts and osteoclasts or matrix was disturbed in the E-64 group.

It is noteworthy that the osteoblast sphericity at R2 was moderately correlated with formation (Fig. 6c). We observed many cuboidal osteoblasts in the resorption sites (Fig. 7d, g). The cuboidal osteoblasts, which are rich in rough endoplasmic reticulum and Golgi apparatus, are deemed as mature osteoblasts which produce bone matrix [35]. We have also reported that cuboidal osteoblasts with high sphericity indicate mature osteoblasts [17]. However, in this study, considering the time points (R2), the location, and morphology, we hypothesized that cuboidal osteoblasts could potentially be reversal cells, a precursor of osteoblasts that promotes the transition from the bone resorption phase to the bone formation phase [36]. The reversal cells also secrete matrix metalloproteinases to remove undigested collagen remnants and prepare the bone surface for subsequent bone formation [36, 37]. Further studies are needed to determine the characteristics of these cells.

Meanwhile, this moderate correlation between formation and osteoblast sphericity almost disappeared in the E-64 group and the emergence of spherical osteoblasts at the resorption site was suppressed, which may be the cause of the loss of the topological and quantitative coordination of matrix resorption and formation (Figs. 6b, c, 7g). Possible explanations for the loss of spherical osteoblasts are as follows. (1) E-64 treatment suppresses the secretion of coupling factors such as Cthrc1 [38] and C3a [39] from osteoclasts directly, or indirectly through the inhibition of cathepsin K leading to some negative feedback effects on osteoclasts. (2) Few collagen remnants that required cleaning by reversal cells were formed in the resorption pits because of the suppression of cathepsin K. This leads to the reduction in the reversal cells appearing at the resorption sites. (3) E-64 suppressed the expression and/or function of receptors for coupling factors such as S1P and ephrin B2 in osteoblasts [40]. A determination of which of these possibilities is correct will contribute to our understanding of the mechanisms of coupling.

A major issue in this study is whether these observations in our system reflect in vivo phenomena. Few studies have evaluated the effect of wide-spectra cysteine proteinase inhibitors in vivo [41, 42]. For example, the administration of E-64 suppressed lipopolysaccharide-induced bone resorption [42]. In contrast, the effects of specific inhibition for cathepsin K have been studied in several species of animals. An osteoclast-specific deletion of cathepsin K suppressed bone resorption and increased the formation of cancellous bone [10]. The cathepsin K inhibitor SB-553484 suppressed the resorption of cancellous bone and promoted the formation of cortical bone [43].

ODN suppressed the bone turnover of the lumbar spine of Rhesus monkeys while maintaining the osteoclast number, and increased bone mineral density [11]. ODN treatment reduced the trabecular and intracortical bone formation rate (BFR) and increased the endocortical BFR and periosteal BFR in femurs [44]. The cathepsin K inhibitor ONO-5334 and alendronate preferentially increased the cortical bone mass and the trabecular bone mass, respectively [45].

In human studies, ODN reduced both resorption and formation markers [46]. Histomorphometric analyses revealed that ODN reduced osteoblast parameters and eroded surfaces but increased the osteoclast number later [47]. Together the above-cited studies suggested that the inhibition of cathepsin K suppresses bone resorption and subsequent formation in the cancellous bones to some extent while promoting bone formation in the cortical bones. In this study, E-64 suppressed both resorption and formation to a lesser extent than ZOL, which does not contradict the in vivo findings. However, few studies have addressed the effect of cathepsin K inhibition on coupling.

Jensen et al. demonstrated that some osteoclasts were detached from the bone surface and shallow resorption pits were increased in ODN-treated animals [23], as this study (Fig. 1c). However, they also observed an increase in the reversal cells and cuboidal osteoblasts by ODN treatment, whereas we observed a loss of spherical osteoblasts in the resorption pits (Figs. S6a, 6a, 7f, g). The differences in findings may be attributable to the difference in the spectra of E-64 and cathepsin K-specific inhibition, and/or to the animal species used. To confirm whether our system can reproduce the phenomena that occur in vivo, the effects of E-64 on bone remodeling and coupling should be examined in a mouse model.

Another issue is that there is also a difference in oxygen concentration between this in vitro system and the in vivo environment. Mature osteoclast and osteoclast precursors are under an oxygen tension of 5% in vivo, a lower level than that of the atmospheric conditions [48, 49]. Both osteoclasts and osteoblasts behave differently under oxygen tensions of 5% and 21%, as to proliferation, differentiation, and migration [50].

In conclusion, our in vitro system demonstrated not only the quantitative and topological correlation between bone resorption and bone formation, suggesting the existence of coupling, but also the correlations between osteoblasts and osteoclasts and between matrix resorption/formation and these cells. We also observed that the inhibition of cysteine proteinase disrupted the coordination between resorption and formation. Although in vivo evidence remains to be obtained, our present findings provide valuable insights for a deeper understanding of coupling mechanisms and the development of innovative therapies targeting osteoporosis.

### Supplementary Information

Below is the link to the electronic supplementary material.

Supplementary file1 (DOCX 15591 KB)
