# Supplementary material for: Inhibition of cysteine protease disturbs the topological relationship between bone resorption and formation in vitro
Source: J Bone Miner Metab. 2024 Feb 20;42(2):166–84. doi: 10.1007/s00774-023-01489-w (PMC10982105; doi:10.1007/s00774-023-01489-w)
Supplement: Supplementary file 1 — Supplementary file1 (DOCX 15591 KB) [file 774_2023_1489_MOESM1_ESM.docx]

**SUPPLEMENTARY INFORMATION**

**Inhibition of cysteine protease disturbs the topological relationship between bone resorption and formation in vitro**

Sayaka Ono^1^, Naoki Tsuji^2^, Tomoaki Sakamoto^2^, Shuya Oguchi^3^, Takashi Nakamura^4^, Kazuto Hoshi^1,2,3^ & Atsuhiko Hikita^2,*^

^1^Department of Sensory and Motor System Medicine, Graduate School of Medicine, The University of Tokyo, Tokyo, 113-8655, Japan. ^2^Department of Tissue Engineering, The University of Tokyo Hospital, Tokyo, 113-8655, Japan. ^3^Department of Oral-Maxillofacial Surgery, and Orthodontics, The University of Tokyo Hospital, Tokyo, 113-8655, Japan. ^4^Department of Biochemistry, Tokyo Dental College, Tokyo, 101-0061, Japan. *email: ahikita-tky@g.ecc.u-tokyo.ac.jp

**Supplementary Figure S1. a:** Fluorescence imaging and ALP staining of osteoblasts isolated from EGFP mice. **b:** Fluorescence imaging and TRAP staining of osteoclasts differentiated from bone marrow cells of Ctsk-Cre × ROSA26-tdTomato mice.

**Supplementary Figure S2.** Optimization of the concentrations of E-64 and zoledronic acid monohydrate (ZOL). **a:** Optimization for E-64. *Upper panels:* Bright-field images for day 14 resorption pits observed as white spots. *Lower panels:* Merged images of phase contrast and fluorescence microscopy for osteoclasts differentiated from bone marrow cells of Ctsk-Cre × ROSA26-tdTomato mice day 5. The osteoclast precursor cells were plated in calcium phosphate-coated plates and stimulated with RANKL and M-CSF. **b:** Quantification of the percentage of pit area at each concentration on day 14. n = 12**. c:** Quantification of the percentage of tdTomato-positive area at each concentration on day 4-5, relative to 0 µM in each lot. n=3. There was no significant difference. **d:** Optimization for ZOL. *Upper panels:* Merged images of phase contrast and fluorescence microscopy for osteoclasts differentiated from bone marrow cells of Ctsk-Cre × ROSA26-tdTomato mice. *Lower panels:* TRAP staining for osteoclasts. Scale bars: 250 µm. **e:** Quantification of the number of TRAP-positive cells with actin lings per one field of view, relative to 0 µM in each lot. n=3. *P = 0.0432. **b, c, e:** Data are mean ± SE and were analyzed by the Kruskal-Wallis test followed by the Steel-Dwass test.

**Supplementary Figure S3. a:** TRAP staining of osteoclasts differentiated from bone marrow cells of Ctsk-Cre × ROSA26-tdTomato mice. **b:** Quantification of the number of TRAP-positive cells per well. n=24. **c:** Staining of the nuclei of osteoclasts by Hoechst 33342. **d:** Quantification of the number of nuclei per osteoclast. Control: n=24, E-64: n=21, ZOL: n=22. **e:** Alizarin red S staining of calcified nodules after 28 days of differentiation of osteoblasts isolated from EGFP mice. **f:** Absorbance of alizarin red S at 450 nm. n=9. **b, d, f:** Data are mean ± SE and were analyzed by the Kruskal-Wallis test followed by the Steel-Dwass test. **g-n:** Uptake of 6-FAM-ZOL in the in vitro reconstruction system. 6-FAM-ZOL was added to the co-culture medium at a concentration of 1 µM for 2 weeks instead of normal ZOL. To enhance image visibility, adjustments using look-up tables (LUTs) with consistent values were applied to images captured at each magnification. **g-j:** Control. **k-n:** 6-FAM-ZOL. **g, k:** Volumetric views of 3D data acquired by 2-photon microscopy at R2. Maximal impression images displayed by IMARIS are presented. *Gray:* Second harmonic generation (SHG; collagen), *Green:* EGFP (osteoblasts), *Red:* tdTomato (osteoclasts), *Yellow:* 6-FAM-ZOL. **h, l:** Orthogonal views of osteoclasts from panels **g** and **k**, as indicated by *blue boxes*. **i, m:** Cropped images from panels h and i, respectively, as indicated by *yellow boxes*. **j, n:** Surface rendering images of **i** and **m** displayed by IMARIS. The green channel outside of osteoclasts was masked. *Red:* tdTomato (osteoclasts), *Green:* 6-FAM-ZOL inside osteoclasts.

~~~~

**Supplementary Figure S4.** Analysis of the correlations between the matrix changes and the osteoclast volume. **a:** Changes in the osteoclast volume of each ROI over time. Each *dot* indicates the sum of the tdTomato volume for each ROI. Control: n=240, E-64: n=256, ZOL: n=272.

**Supplementary Figure S5.** Analysis of the correlations between matrix changes and the EGFP volume. **a:** Changes in the osteoblast volume of each ROI over time. Each *dot* indicates the sum of EGFP volume for each ROI. Control: n=240, E-64: n=256, ZOL: n=272.

**Supplementary Figure S6.** Analysis of the correlations between bone resorption/formation and osteoblast sphericity. **a:** Changes in the osteoblast sphericity of each ROI over time. Each *dot* indicates the sum of the EGFP sphericity for each ROI.

**SUPPLEMENTARY MATERIALS AND METHODS**

**Preparation of primary osteoblasts**

Primary osteoblasts were isolated from EGFP mice as described [1, 2, 3, 4] with a few modifications. Briefly, the calvaria were harvested from newborn mice aged 0–5 days, and the soft tissues attached to their surface were removed with cell scrapers. Parietal bones were collected and incubated in 3.5 mL of Minimum Essential Medium-α (MEM-α; Thermo Fisher Scientific) supplemented with 100 µg/mL of collagenase P (Roche Diagnostics), and 88 µL of 0.05% trypsin/EDTA (Thermo Fisher Scientific) per calvaria at 37°C for 20 min with a shaking water bath. The parietal bones were then chopped into small pieces and incubated in 800 µL MEM-α supplemented with 200 µg/mL collagenase P and 20 µL of 0.05% trypsin/EDTA per calvaria for 15 min at 37°C.

After the incubation, enzyme was removed, and the bone pieces were washed twice with 4 mL of MEM-α supplemented with 15% fetal bovine serum (FBS), 100 U/mL penicillin (Sigma-Aldrich), and 100 µg/mL streptomycin (Sigma-Aldrich) per calvaria. The bone pieces were cultured with a new culture medium (MEM-α supplemented with 15% FBS, 100 U/mL penicillin, and 100 µg/mL streptomycin) for 5–6 days, and cells that grew out of the bone pieces were collected.

To confirm whether osteoblasts were harvested adequately, we cultured 6.0 × 10^4^ osteoblasts from EGFP mice in one well of six-well plates for 2 days with osteoblast differentiation medium [MEM-α supplemented with 10% FBS, 100 U/mL penicillin, 100 µg/mL streptomycin, 100 µg/mL L(+)-ascorbic acid (Fujifilm Wako), and 5 mM β-glycerophosphate disodium salt hydrate (Sigma-Aldrich)] with 0.1 µM cFMS Receptor Inhibitor II (Santa Cruz Biotechnology). The cFMS Receptor Inhibitor II was supplemented to counteract any potential effects of macrophage lineage cells contaminated in the primary osteoblasts [5]. The TRAP/ALP Stain Kit (Fujifilm Wako) was used to stain osteoblasts with alkaline phosphatase (ALP) activity per the manufacturer's instructions. The fluorescence of the cells was observed with a BZ-9000 fluorescence microscope (Keyence) before staining and in a bright field after staining.

**Preparation of bone marrow macrophages**

Bone marrow cells were collected from the tibia, femur, and humerus of Ctsk-Cre × ROSA26-tdTomato mice as described [1, 3, 6, 7, 8]. The bone marrow cavity was flushed with phosphate-buffered saline (PBS), and the solution was centrifuged at 430*g* for 5 min for the collection of the bone marrow cells. Cells were treated with Tris-NH_4_Cl for 2 min to lyse red blood cells and then centrifuged at 430*g* for 3 min after the addition of 1 mL of FBS [9]. After an overnight culture with MEM-α supplemented with 10% FBS, 100 U/mL penicillin, 100 µg/mL streptomycin, and 10 ng/mL macrophage colony-stimulating factor (M-CSF) (R&D Systems), floating cells were collected.

To determine whether cells possessing the ability to differentiate into osteoclasts were harvested successfully, we cultured bone marrow macrophages from Ctsk-Cre×ROSA26-tdTomato mice at a concentration of 4.0 × 10^5^ cells/well in a six-well plate, with MEM-α containing 10% FBS, 100 U/mL penicillin, 100 g/mL streptomycin, and 30 ng/mL M-CSF for 1 day, and with MEM-α supplemented with 10% FBS, 100 U/mL penicillin, 100 µg/mL streptomycin, 30 ng/mL M-CSF, 100 ng/mL RANKL (receptor activator of nuclear factor kappa-Β ligand) (Fujifilm Wako), and GlutaMax™ (Gibco) for 7 days. The TRAP/ALP Stain Kit was used to stain osteoclasts with tartrate-resistant acid phosphatase (TRAP) activity per the manufacturer's instructions. Cells were observed with a BZ-9000 fluorescence microscope for fluorescence before staining and in a bright field after staining.

**Determination of the E-64 concentration**

Bone marrow macrophages from Ctsk-Cre × ROSA26-tdTomato mice were seeded to a Bone Resorption Assay Plate 24 (PG Research, Tokyo, Japan) at a concentration of 2.0 × 10^5^ cells/well. Cells were cultured with MEM-α supplemented with 10% FBS, 100 U/mL penicillin, 100 µg/mL streptomycin, and 10-30 ng/mL M-CSF for 1-2 days, and with MEM-α supplemented with 10% FBS, 100 U/mL penicillin, 100 µg/mL streptomycin, 10-30 ng/mL M-CSF, 100 ng/mL RANKL, and GlutaMax with 0, 5, 10, 20 or 40 µM of E-64 (Peptide Institute) for 14 days. Cells and resorption pits were observed with a DMi8 fluorescence microscope (Leica Microsystems).

**Determination of the zoledronic acid concentration**

Bone marrow macrophages isolated from Ctsk-Cre × ROSA26-tdTomato mice were seeded to a 24-well plate at a concentration of 2.0 × 10^5^ cells/well. The cells were incubated with MEM-α supplemented with 10% FBS, 100 U/mL penicillin, 100 µg/mL streptomycin, and 10 ng/mL M-CSF for 2 days, and with MEM-α supplemented with 10% FBS, 100 U/mL penicillin, 100 µg/mL streptomycin, 25 ng/mL M-CSF, 100 ng/mL RANKL, and GlutaMax with 0, 0.5, 1, 2 or 4 µM of Zoledronic Acid Monohydrate (Fujifilm Wako) for 5 days. The TRAP/ALP Stain Kit was used to stain the cells per the manufacturer's instructions. The cells were observed with the DMi8 fluorescence microscope.

**Osteoclast differentiation assay**

Bone marrow macrophages from Ctsk-Cre × ROSA26-tdTomato mice were seeded to a 96-well plate at a concentration of 1.7 × 10^4^ cells/well. The cells were incubated with MEM-α supplemented with 10% FBS, 100 U/mL penicillin, 100 µg/mL streptomycin, and 30 ng/mL M-CSF for 2 days. The cells were next cultured with MEM-α supplemented with 10% FBS, 100 U/mL penicillin, 100 µg/mL streptomycin, 10 ng/mL M-CSF, 100 ng/mL RANKL, and GlutaMax with or without 10 µM E-64 or 1 µM ZOL for 5 days. The TRAP/ALP Stain Kit was used to stain the cells per the manufacturer's instructions. The cells were observed with the DMi8 fluorescence microscope in a bright field. Stained cells were counted manually.

**Osteoblast differentiation assay**

Osteoblasts from EGFP mice calvariae were seeded in 24-well plates at 4.5 × 10^4^ cells/well and cultured with MEM-α supplemented with 15% FBS, 100 U/mL penicillin, and 100 µg/mL streptomycin with or without 10 µM E-64 or 1 µM ZOL. After 3 days, when the cells reached confluence, they were cultured with osteoblast differentiation medium supplemented with 0.1 µM cFMS Receptor Inhibitor II with or without 10 µM E-64 or 1 µM ZOL. After 28 days, the matrix was stained with 1% alizarin red S solution (Muto Pure Chemicals) for 10 min and rinsed with PBS per the manufacturer's instructions. Cell images were taken with a digital camera. The matrix was dissolved in 5% formic acid, and the absorbance of the supernatant was measured with a 2030 Multilabel Reader ARVO X3 (PerkinElmer) at 450 nm for 1.0 sec.

**Evaluation of osteoclast multinucleation**

Bone marrow macrophages from Ctsk-Cre × ROSA26-tdTomato mice were seeded in a 24-well plate at a concentration of 1.0 × 10^5^ cells/well. The cells were cultured with MEM-α supplemented with 10% FBS, 100 U/mL penicillin, 100 µg/mL streptomycin, and 30 ng/mL M-CSF for 2 days. The cells were then cultured with MEM-α supplemented with 10% FBS, 100 U/mL penicillin, 100 µg/mL streptomycin, 10 ng/mL M-CSF, 100 ng/mL RANKL, and GlutaMax with or without 10 µM E-64 or 1 µM ZOL for 5 days. Next, the cells were stained with 5 µg/mL of Cellstain^®^-Hoechst 33342 solution (Dojindo) diluted in PBS for 5 min at room temperature per the manufacturer's protocol. The cells were observed with the DMi8 fluorescence microscope, and stained nuclei were counted manually.

**Cell culture for the in vitro reconstruction system for a bone cell network**

Osteoblasts from EGFP mice were cultured in 60-mm dishes at 4 × 10^5^ to 5.5 × 10^5^ cells/dish with MEM-α supplemented with 15% FBS, 100 U/mL penicillin, and 100 µg/mL streptomycin until they reached confluence at 3–5 days. For the induction of osteoblast differentiation, the cells were cultured in the osteoblast differentiation medium supplemented with 0.1 µM cFMS Receptor Inhibitor II. To ensure that there were no differences in the cultured osteoblasts between the control, E-64, and ZOL groups, the osteoblasts isolated on the same day for each series were effectively suspended and evenly seeded from the same cell suspension. Calcified nodules were formed after 4–6 weeks of differentiation culture, and 2 × 10^6^ bone marrow macrophages from Ctsk-Cre×ROSA26-tdTomato mice were added. They were co-cultured with the co-culture medium (MEM-α supplemented with 10% FBS, 100 U/mL penicillin, 100 µg/mL streptomycin, 10^−6^ M prostaglandin E2 [Sigma-Aldrich], and 10^−8^ M 1α, 25-dihydroxy vitamin D3 [Sigma-Aldrich] with or without 10 µM E-64 or 1 µM ZOL) for 2 weeks, and with osteoblast differentiation medium with or without 10 µM E-64 or 1 µM ZOL for 3 weeks. After the osteoblast differentiation, E-64 or ZOL were continuously administered to the co-culture medium of bone marrow cells. Fresh drug solutions were exchanged with each medium change.

To observe the uptake of ZOL in the in vitro reconstruction system, a fluorescent carboxyphosphonate imaging reagent, 6-FAM-ZOL (BioVinc), were continuously administered to the co-culture medium at a concentration of 1 µM instead of normal ZOL for 2 weeks.

**References**

1. Hikita A, Iimura T, Oshima Y, et al (2015) Analyses of bone modeling and remodeling using in vitro reconstitution system with two-photon microscopy. Bone (New York, NY) 76:5–17. https://doi.org/10.1016/j.bone.2015.02.030

2. Tsuji N, Sakamoto T, Hoshi K, Hikita A (2022) Spatiotemporal Analysis of Osteoblast Morphology and Wnt Signal‐Induced Osteoblast Reactivation during Bone Modeling in Vitro. JBMR Plus 6

3. Oguchi S, Sakamoto T, Hoshi K, Hikita A (2022) Quantitative analyses of matrices, osteoblasts, and osteoclasts during bone remodeling using an in vitro system. J Bone Miner Metab. https://doi.org/10.1007/s00774-022-01381-z

4. Inose H, Ochi H, Kimura A, et al (2009) A microRNA regulatory mechanism of osteoblast differentiation. Proc Natl Acad Sci 106:20794–20799

5. Ohno H, Kubo K, Murooka H, et al (2006) A c-fms tyrosine kinase inhibitor, Ki20227, suppresses osteoclast differentiation and osteolytic bone destruction in a bone metastasis model. Mol Cancer Ther 5:2634–2643

6. Fukuda T, Takeda S, Xu R, et al (2013) Sema3A regulates bone-mass accrual through sensory innervations. Nature 497:490–493

7. Fujita K, Iwasaki M, Ochi H, et al (2012) Vitamin E decreases bone mass by stimulating osteoclast fusion. Nat Med 18:589–594

8. Chevalier C, Çolakoğlu M, Brun J, et al (2021) Primary mouse osteoblast and osteoclast culturing and analysis. STAR Protoc 2:100452. https://doi.org/10.1016/j.xpro.2021.100452

9. Matsumoto M, Sudo T, Saito T, et al (2000) Involvement of p38 mitogen-activated protein kinase signaling pathway in osteoclastogenesis mediated by receptor activator of NF-κB ligand (RANKL). J Biol Chem 275:31155–31161
